# Comparative physiological and transcriptomic analyses provide insights into fruit softening in Chinese cherry [*Cerasus pseudocerasus* (Lindl.) G.Don]

**DOI:** 10.3389/fpls.2023.1190061

**Published:** 2023-07-17

**Authors:** Yan Wang, Lan Ma, Yan Ma, Tai Tian, Jing Zhang, Hao Wang, Zhenshan Liu, Qing Chen, Wen He, Yuanxiu Lin, Yunting Zhang, Mengyao Li, Shaofeng Yang, Yong Zhang, Ya Luo, Haoru Tang, Xiaorong Wang

**Affiliations:** ^1^ College of Horticulture, Sichuan Agricultural University, Chengdu, Sichuan, China; ^2^ Institute of Pomology and Olericulture, Sichuan Agricultural University, Chengdu, Sichuan, China; ^3^ Key Laboratory of Agricultural Bioinformatics, Ministry of Education, Chengdu, Sichuan, China

**Keywords:** Chinese cherry (*Cerasus pseudocerasus* (Lindl.) G.Don), fruit softening, cell wall, transcriptome, phytohormones, transcription factors

## Abstract

Fruit softening is a complex, genetically programmed and environmentally regulated process, which undergoes biochemical and physiological changes during fruit development. The molecular mechanisms that determine these changes in Chinese cherry [*Cerasus peseudocerasus* (Lindl.) G.Don] fruits are still unknown. In the present study, fruits of hard-fleshed ‘Hongfei’ and soft-fleshed ‘Pengzhoubai’ varieties of Chinese cherry were selected to illustrate the fruit softening at different developmental stages. We analyzed physiological characteristics and transcriptome profiles to identify key cell wall components and candidate genes related to fruit softening and construct the co-expression networks. The dynamic changes of cell wall components (cellulose, hemicellulose, pectin, and lignin), the degrading enzyme activities, and the microstructure were closely related to the fruit firmness during fruit softening. A total of 6,757 and 3,998 differentially expressed genes (DEGs) were screened between stages and varieties, respectively. Comprehensive functional enrichment analysis supported that cell wall metabolism and plant hormone signal transduction pathways were involved in fruit softening. The majority of structural genes were significantly increased with fruit ripening in both varieties, but mainly down-regulated in Hongfei fruits compared with Pengzhoubai, especially DEGs related to cellulose and hemicellulose metabolism. The expression levels of genes involving lignin biosynthesis were decreased with fruit ripening, while mainly up-regulated in Hongfei fruits at red stage. These obvious differences might delay the cell all degrading and loosening, and enhance the cell wall stiffing in Hongfei fruits, which maintained a higher level of fruit firmness than Pengzhoubai. Co-expressed network analysis showed that the key structural genes were correlated with plant hormone signal genes (such as abscisic acid, auxin, and jasmonic acid) and transcription factors (MADS, bHLH, MYB, ERF, NAC, and WRKY). The RNA-seq results were supported using RT-qPCR by 25 selected DEGs that involved in cell wall metabolism, hormone signal pathways and TF genes. These results provide important basis for the molecular mechanism of fruit softening in Chinese cherry.

## 1 Introduction

Chinese cherry [*Cerasus pseudocerasus* (Lindl.) G.Don], as an economically fruit crop native to China, is characterized by the characteristic of precocity, high ornamental value, delicious taste, and rich nutrition (Yü et al., 1986; Chen et al., 2016). Recently, the cultivation of Chinese cherry has been growing vigorously in China, which has increasingly contributed to rural tourism and rural revitalization especially South China (Chen et al., 2016). However, the fruits are susceptible to softening and rotting after harvest, strongly limiting the circulation to the consumer market, which has greatly hindered the development of Chinese cherry industry (Wang et al., 2022c). Therefore, uncovering the mechanisms behind softening is a requirement before attempting to resolve this constraint in Chinese cherry.

Texture change of most fleshy fruits during ripening is largely caused by softening, due to the changes in ultrastructure and metabolism of cell wall (Brummell, 2006; Wang et al., 2018; Peng et al., 2022). The cell wall polysaccharides, containing cellulose, hemicellulose, pectin, and lignin are hydrolyzed during fruit ripening and softening process, resulting in the loss of cell wall structure and the reduction of intercellular adhesion (Santiago-Domenech et al., 2008). These processes are regulated by a series of cell wall-modification enzymes (Brummell et al., 2004). Pectinases such as polygalacturonase (*PG*), pectin methylesterase (*PME*), pectate lyase (*PL*), and β-galactosidase (*BGAL*) catalyze different forms of pectin, resulting in the changes in pectin solubilization. Three types of cellulases, including endo-glucanase (*EGase*), cellobiohydrolase (*CBH*), and β-glucosidase (*BGLU*), were involved in cellulose decomposition. Xyloglucan endotransglucosylase/hydrolase (*XTH*) promotes the depolymerization of hemicellulose, and expansin (*EXP*) participates in fruit cell wall loosening. Phenylalanine ammonia lyase (*PAL*), cinnamyl CoA reductase (*CCR*), and peroxidase (*POD*) play essential roles in the biosynthesis of lignin (Cai et al., 2006). The expression profiles of the genes related to cell wall metabolism is significantly correlated with fruit ripening and softening (Zhang et al., 2017; Moya-Leon et al., 2019; Zhai et al., 2021; Peng et al., 2022). Modulating the expression of these genes affects the fruit firmness and postharvest shelf life in hawthorn (Xu et al., 2016), strawberry (Quesada et al., 2009; Witasari et al., 2019; Xue et al., 2020), and sweet cherry (Zhai et al., 2021; Zhai et al., 2022). The correlation between cell wall degradation enzymes and fruit firmness has been described in many fleshly fruits, while the underling mechanisms of fruit softening in Chinese cherry have not been involved so far.

Plant hormones, including ABA (abscisic acid), ETH (ethylene), IAA (auxin), BR (brassinosteroid), GA (gibberellin acid), JA (jasmonic acid), and SA (salicylic acid), play important roles in fruit ripening and softening (Peng et al., 2022). ABA has been considered to be a central trigger for the ripening of non-climatic fruits (Wang et al., 2022b). ABA-induced fruit ripening has been reported in many fruits, such as sweet cherry (Zhai et al., 2022), grape (Pilati et al., 2017), and strawberry (Luo et al., 2020). ABA modulates fruit softening in the upstream signaling pathway of ethylene (Diretto et al., 2020), or induces changes in the transcription of genes encoding cell wall-degrading enzymes (Li et al., 2015). In peach, *PpIAA1* can activate the expression of *PpACS1* by directly binding to its promoter (Wang et al., 2021). Work on several fruits has supported that BR and JA promote fruit ripening (Chai et al., 2013; Li et al., 2017b), while SA inhibits ripening (Zhang et al., 2003).

Transcription factors (TFs) have also been demonstrated to play roles in fruit softening by regulating the expression of cell wall modifying genes, such as MADS-Box, MYB, AP2/ERF, NAC, WRKY, BZR, and bZIP, etc (Peng et al., 2022). PpERF4 in peach can bind to the promoters of *PpACO1* and *PpIAA1* genes to activate their transcription levels (Wang et al., 2021). PrupeSEP1 could interact with promoter of *PrupePG2* and *PrupePG3* to regulate fruit ripening and softening of melting flesh peaches (Li et al., 2017a). In strawberry, FcNAC1 can enhance the expression of *FcPL* by binding to its promoter, which is involved in pectin metabolism (Carrasco-Orellana et al., 2018). Banana MaBZR1/2 could inhibit ETH biosynthesis and fruit softening by directly repressing *MaPL2*, *MaEXP2*, and *MaXET5* (Shan et al., 2020). In sweet cherry, PaMADS7 directly interacts with the promoter of *PaPG1* to affect fruit softening (Qi et al., 2020), and C_2_C_2_-type TFs PavDOF2/6/15 could bind directly to the promoters of genes encoding cell wall-degrading enzymes to activate or repress their transcription (Zhai et al., 2022).

In the present study, we compared two Chinese cherry varieties with different fruit firmness characters by physiological characteristics, which revealed that cellulose, hemicellulose, pectin and lignin are povital for maintaining cell wall integrity and fruit softening. Then, the differentially expressed genes (DEGs) involved in fruit softening at key developmental stages were identified through transcriptomic analysis. The weighted gene co-expression network (WGCNA) analysis was used to screen key genes associated with fruit softening in Chinese cherry and construct the gene regulatory network. Our study will provide insights into the genetic basis of fruit softening and provide useful information for genetic improvement of Chinese cherry.

## 2 Materials and methods

### 2.1 Plant materials

**Figure 1:**
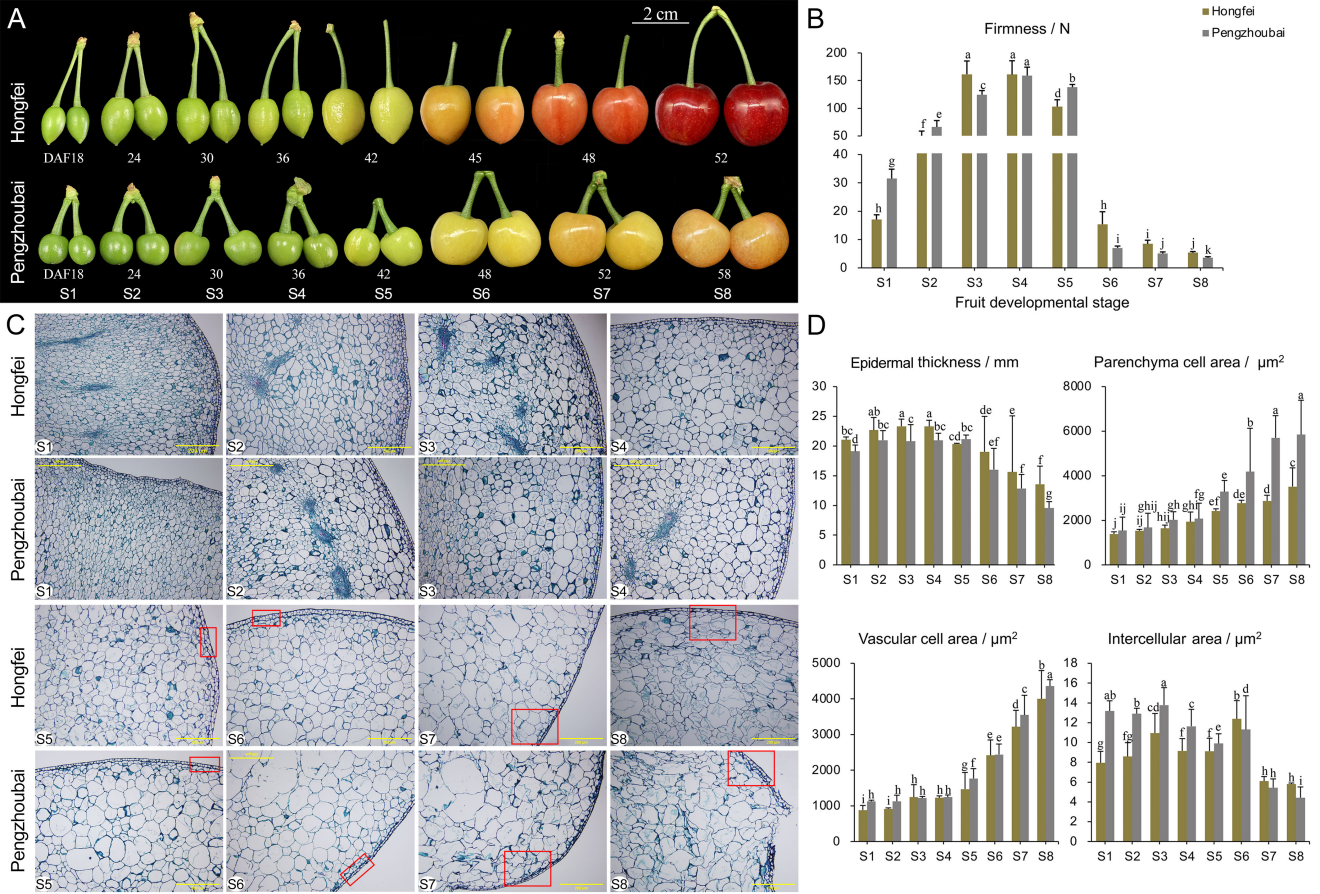
Changes in firmness and microstructure of Chinese cherry fruits. **(A)** Phenotypes during fruit development for Hongfei and Pengzhoubai. Fruit development was divided into eight stages according to our field investigation. DAFB, days after full bloom. **(B)** Dynamic changes of firmness during fruit development in Hongfei and Pengzhoubai. **(C)** Paraffin sections of the fruit flesh. The red rectangles represent the differences between the two varieties. Scale bar indicates 200 μm at 100 × magnification. **(D)** Dynamic changes of microstructure during fruit development in Hongfei and Pengzhoubai. In **(B, D)**, data are shown as means ± standard deviation (SD) of three biological replicates. Different letters indicate statistically significant difference by Duncan’s multiple range test (*P* < 0.05).

The plant materials were grown under field conditions at Cherry Germplasms Resources of Sichuan Province, China. Based on the comprehensive evaluation of fruit texture of 50 representative Chinese cherry samples, two varieties, Hongfei and Pengzhoubai, were selected because of their firmness difference (Ma et al., 2023). Fruits with the uniform size, same appearance, and no scars were sampled based on the fruit phenology (**Figure 1A**). Ten cherries were used for firmness measurement, and the other forty cherries were frozen in liquid nitrogen, and stored at -80°C immediately.

### 2.2 Measurement of fruit firmness and microstructure

Fruit firmness was evaluated by texture profile analysis (TPA) using the TMS-Pilot texture analyzer (TL-Pro testing system, FTC, Atlanta, USA). Each cherry was compressed in the equatorial position by a P/50 (50 mm) flat cylindrical probe. The measurement was conducted according to the parameters with test speed of 50 mm·min^−1^, compression degree of 25% in 5 s, and trigger force of 0.375 g. Firmness is regarded as the peak force during the first compression of the fruits (Liu et al., 2017), with three biological replicates.

The paraffin section was made and analyzed as described by Han et al. (2016a). The samples were observed, photographed, and analyzed using an Olympus BX51 microscope (Olympus, Tokyo, Japan). The epidermal thickness, parenchyma cell area, vascular cell area, and intercellular area for cells were measured by the IPP6.0 (Image Pro Plus) software. Thirty cells were measured for each slide per sample point.

### 2.3 Extraction and measurement of cell wall components and degrading enzyme activities

The extraction of cell wall components, including water-soluble pectin (WSP), covalent-soluble pectin (CSP), ionic-soluble pectin (ISP), and cellulose, hemicellulose following the methods according to Zhai et al. (2021). Briefly, 5.0 g of freeze-dried fruit tissues were grounded into powder in liquid nitrogen and homogenized in 80% ethanol before boiling for 20 min. After rapid cooling, the homogenate extracts were centrifuged for 20 min at 12,000× g at 4°C. The pellets were sequentially washed in 80% ethanol and in pure acetone. The final insoluble component was dried overnight at 45°C to constant weight as cell wall material (CWM, mg·g^−1^). The pectin content was quantified by the m-hydroxydiphenol method (Blumenkrantz and Asboe-Hansen, 1973) using galacturonic acid as a standard. The cellulose and hemicellulose contents was determined by the anthrone colorimetric method (Dische, 1962) with glucose as standards. Lignin content was determined as described by Cai et al. (2006).

Cellulase enzyme activity was analyzed following the methods reported by Deng et al. (2005). PG and PME activities were determined using the available commercial test kits (Suzhou Grace Biotechnology Co., Ltd., Suzhou, China) based on the manufacturer’s recommendations. The β-Gal enzyme’s activity was estimated with Wei et al. (2010)’s method. The detailed protocol of Ji et al. (2021) was employed for activity determination of β-Glu enzyme.

### 2.4 Determination of endogenous hormones

To check the levels of endogenous hormones during fruit development, fruit samples were collected at S3, S5, and S7 stages for two varieties. Abscisic acid (ABA), auxin (indole-3-acetic acid, IAA), ethylene, and JA (jasmonic acid) were extracted by abundant cold methanol (80%, v/v). Crude extract was condensed by vacuum evaporation and hormones were re-extracted by ethyl acetate at pH 3.0 (He et al., 2022). The content of four types of phytohormones were measured using enzyme-linked immunosorbent assay (ELISA, Phytohormone Research Institute, Nanjing Agricultural University, Nanjing, China), following the manufacturer’s instructions.

### 2.5 Transcriptome analysis

Eighteen libraries sampled from three developmental stages (S3, S5, and S7) of Hongfei and Pengzhoubai were constructed. RNA-seq was performed on the MGI2000 platform with an average depth of 30×. The raw sequences have been subjected to the CNGB database under the project number CNP0003682 (https://db.cngb.org/cnsa/experiment/page/batch/sub037808/view/). The clean reads were mapped to the Chinese cherry reference genome (unpublished) by HISAT2 v.2.1.0. The number of reads mapped to each gene was count using HTSeq v.0.6.1. The FPKM (fragments per kilobase per million base pairs) value for each gene was calculated by analyzing the gene length and reads mapped to the gene.

DEGs were screened in pair-wise comparisons using the EdegR package. Genes were defined as significantly and differentially expressed with a |log_2_fold-change| > 2 and *P* value < 0.05. Fuzzy cMeans clustering method was used to visualize gene expression patterns using R package (v.1.4.1717). Normalized expression values of genes were calculated using Z scores based on input FPKM values of all samples. TBtools software (v.1.106) (Chen et al., 2020) was used to test for enrichment of DEGs in GO (gene ontology) and KEGG (Kyoto Encyclopedia of Gene and Genomes) enrichment pathways (*P* < 0.05). The heatmap based on gene expression levels shown as log_2_(FPKM + 1) was visualized by TBtools.

The PlantTFDB online tool v.5.0 (http://planttfdb.cbi.pku.edu.cn/, accessed on 26 January 2023) (Jin et al., 2017) was used to screen and categorize the predicted possible transcription factors from DEGs. For correlation network analysis, a Pearson correlation evaluation was carried out using the FPKM values from all samples. The co-expressed genes were identified by the criterion of |r| ≥ 0.90 and *P*-value ≤ 0.05. The interactive networks among structural genes, plant hormone signaling genes, and TF genes involved in fruit softening were revealed by Cytoscape software (v.3.9.1) (Shannon et al., 2003).

All the DEGs were filtered to remove genes that had low expression levels (FPKM < 2) in all samples. Weighted gene co-expression network analysis was performed in R using default parameters. An adjacency matrix was constructed based on the normalized FPKM values. The phenotype data during fruit softening were imported into the WGCNA R software package to calculate correlation-based connections between phenotypes and modules. Subsequently, the adjacency matrix was converted into a topological overlap matrix (TOM). The genes with identical expression patterns were assigned into one module according to the network, and eigengenes were determined for these modules. The network of the genes from Turquoise and Blue modules was exported using Cytoscape software.

### 2.6 RT-qPCR analysis

To validate the reliability of the transcriptome data, 25 DEGs (**Supplementary Table 1**) were selected for RT-qPCR analysis with three independent biological replicates for each sample. A TIANGEN kit (Beijing, China) assay was used for the extraction of total RNA. The cDNA was synthesized using PrimeScript™ RT-PCR Kit (Takara, Japan). Primer design and RT-qPCR procedure were conducted as described previously (Wang et al., 2023). The relative gene expression level of each gene was calculated by the 2^−△△Ct^ method, with cherry *actin* and *ubiquitin* as internal controls.

### 2.7 Statistical analysis

The physiological indexes were calculated by Microsoft Excel 2020. All the data were shown as the mean ± standard deviation (SD) of the three biological replicates. The significant differences between developmental stages and varieties were analyzed by the Duncan’s multiple range test (*P* < 0.05).

## 3 Results

### 3.1 Changes in firmness and microstructure of Chinese cherry fruits

Based on our previous field observation and firmness measurement (Ma et al., 2023), we selected two varieties Hongfei and Pengzhoubai as materials. Hongfei had the relative firmer fruits, while Pengzhoubai was characterized by the softest fruits. Thus, we measured the dynamic changes in fruit firmness for both varieties at eight developmental stages (**Figure 1A**). Both of them showed an increasing trend in fruit firmness during the early stages (S1-S4) (**Figure 1B**). Firmness gradually decreased with fruit ripening, reaching values at the S5 stage about two thirds that of the S3 stage. The results indicated that the S5 stage is a critical stage between fruit ripening and softening in Chinese cherry. Obviously, fruit firmness of Pengzhoubai declined faster than Hongfei after the S5 stage.

We further observed the cytological characteristics of fruit in the two varieties in paraffin sections. Similar cellular structure was detected for them at the early stage (S1-S4), which was characterized by small and intact cell arrangement (**Figure 1C**). With fruit ripening and softening, the cellular volume expands simultaneously with an increase of the intercellular space due to the degradation of cell wall and middle lamella. Notably, compared to the compact cell structure at the S7 stage observed in Hongfei, we inspected numerous discontinuities in cell wall in Pengzhoubai, indicating that the maintenance of cell wall integrity is compromised.

The microstructure parameters were also determined to confirm the differences between the two varieties (**Figure 1D**). The epidermal thickness slightly increased during the early stage, and steadily decreased from S4 stage. As predicted, Hongfei fruits had larger epidermal thickness than that in Pengzhoubai fruits through developmental stages. The cell area of both parenchyma and vascular increased during fruit development, and became faster from the S5 stage, while the intercellular area continuously declined through fruit developmental stages. All the three parameters in Hongfei fruits were significantly lower than that in Pengzhoubai fruits. Therefore, the Hongfei fruits with relative high firmness is characterized as large epidermal thickness, and small and tight parenchyma cell, vascular cell, and intercellular area.

### 3.2 Changes in cell wall components and degrading enzyme activities during fruit development

We observed the alterations in cell wall components at S3, S5, and S7 stages in both two varieties. As shown in **Table 1**, the contents of cellulose and hemicellulose decreased during fruit development in Chinese cherry, WSP and ISP level increased through the same stage. Obvious differences were detected between them, as hard-fleshed Hongfei fruits included more cellulose, hemicellulose, and less WSP and ISP, than the soft-fleshed Pengzhoubai at three stages. Lignin content in both fruits slightly increased with fruit ripening, and it was significant higher in Hongfei than that in Pengzhoubai through fruit development. These results suggested that fruit softening in Chinese cherry largely attributed to the degradation of cell wall components, and that differences in fruit firmness between varieties were caused by the changes of cellulose, hemicellulose and pectin contents, as well as lignin content.

**Table 1:** Dynamic changes of cell wall components and cell wall degrading enzyme activities in the fruits of Hongfei and Pengzhoubai at three different developmental stages.

Sample	Stage	Cellulose (mg·g^−1^)	Hemicellulose (mg·g^−1^)	CSP (mg·g^−1^)	WSP (mg·g^−1^)	ISP (mg·g^−1^)	Lignin (mg·g^−1^)
Hongfei	S3	44.87 ± 0.49 a	27.59 ± 1.03 a	120.75 ± 5.07 a	56.42 ± 2.14 e	35.97 ± 2.63 e	114.26 ± 9.10 d
	S5	36.93 ± 0.31 c	19.91 ± 2.12 c	114.16 ± 4.72 b	67.31 ± 2.46 d	48.98 ± 2.05 d	135.76 ± 4.22 b
	S7	28.16 ± 0.49 e	13.22 ± 0.60 d	109.69 ± 6.78 b	79.70 ± 2.89 c	95.63 ± 2.04 b	148.49 ± 6.80 a
Pengzhoubai	S3	40.33 ± 0.26 b	24.56 ± 0.10 b	121.43 ± 2.51 a	56.55 ± 1.26 e	34.10 ± 1.16 e	98.83 ± 6.16 e
	S5	32.08 ± 0.35 d	18.84 ± 2.93 c	100.95 ± 1.55 c	90.60 ± 8.09 b	57.53 ± 1.43 c	121.75 ± 7.86 c
	S7	24.86 ± 0.43 f	6.31 ± 0.15 e	90.11 ± 1.79 d	155.58 ± 6.41 a	120.48 ± 12.77 a	135.01 ± 3.81 b
Sample	Stage	Cx (mg·h^−1^·g^−1^)	PG (mg·h^−1^·g^−1^)	PME (μmoL·min^−1^·g^−1^)	β-Gal (nmol·min^−1^·g^−1^)	β-Glu (nmol·min^−1^·g^−1^)	
Hongfei	S3	0.96 ± 0.09 d	0.50 ± 0.04 d	2.98 ± 0.06 a	1.17 ± 0.19 e	7.65 ± 0.65 c	
	S5	0.96 ± 0.06 d	1.27 ± 0.04 c	2.89 ± 0.07 a	2.16 ± 0.19 d	18.20 ± 0.20 b	
	S7	1.92 ± 0.06 a	1.85 ± 0.07 a	2.76 ± 0.04 a	20.01 ± 0.17 a	18.79 ± 0.33 a	
Pengzhoubai	S3	0.57 ± 0.09 e	1.31 ± 0.08 c	0.69 ± 0.17 d	0.53 ± 0.03 f	6.65 ± 0.40 d	
	S5	1.41 ± 0.09 c	1.37 ± 0.11 c	2.22 ± 0.12 b	2.65 ± 0.54 c	17.89 ± 0.19 b	
	S7	1.56 ± 0.08 b	1.47 ± 0.03 b	1.22 ± 0.20 c	16.97 ± 0.36 b	18.22 ± 0.04 b	

CSP, covalent-soluble pectin; WSP, water-soluble pectin, ISP: ionic-soluble pectin; Cx, cellulase; PG, polygalacturonase; PME, pectin methylesterase; β-Gal, β-galactosidase; β-Glu, β-glucosidase. Values are means ± SD based on three biological replicates. Different lowercase letters indicate significant differences, as determined by Duncan’s multiple range test with P < 0.05.

We also determined the activity of cell wall-related enzymes during fruit development and ripening. The enzymatic activity increased with fruit softening in both varieties, while PME activity decreased (**Table 1**). By comparison, Hongfei fruits revealed higher cellulase (Cx), PG, and β-Gal enzyme activity than Pengzhoubai fruits at the three stages. The increase in β-Glu activity was comparable between them. These findings indicated that the cell wall-degrading enzyme activities were closely related to the fruit softening in Chinese cherry.

### 3.3 Changes in plant hormone contents

We further analyzed the dynamic changes in contents of plant hormones during fruit development (**Table 2**). Both ABA and IAA contents kept relative high levels during fruit development, and showed increasing trends with fruit ripening in both varieties. By comparison, ABA level in Hongfei fruits was higher than that in Pengzhoubai fruits at S3 and S5 stage, while it was significantly higher in Pengzhoubai fruits at S7 stage. There was no significant differences for IAA levels at S7 stage between two varieties. The ETH and JA contents slightly increased during fruit ripening, but they kept very low levels through fruit development. These results suggested that ABA and IAA hormones might play important roles with fruit ripening and softening in Chinese cherry.

**Table 2:** Dynamic changes of endogenous hormones in the fruits of Hongfei and Pengzhoubai at three different developmental stages.

Sample	Stage	ABA (ng·g^−1^)	IAA (ng·g^−1^)	ETH (ng·g^−1^)	JA (ng·g^−1^)
Hongfei	S3	613.83 ± 8.06 e	558.91 ± 15.41 d	0.48 ± 0.02 d	0.14 ± 0.01 c
	S5	736.17 ± 9.75 c	649.39 ± 14.46 b	0.55 ± 0.03 c	0.15 ± 0.01 c
	S7	920.01 ± 6.03 b	701.81 ± 13.88 a	0.75 ± 0.02 a	0.17 ± 0.01 b
Pengzhoubai	S3	603.69 ± 9.67 f	553.18 ± 7.12 d	0.43 ± 0.01 e	0.19 ± 0.01 a
	S5	621.04 ± 11.19 d	632.95 ± 9.91 c	0.54 ± 0.05 c	0.15 ± 0.01 c
	S7	977.13 ± 10.34 a	702.61 ± 16.94 a	0.60 ± 0.02 b	0.16 ± 0.01 b

ABA, abscisic acid; IAA, indole-3-acetic acid; ETH, ethylene; JA, jasmonic acid. Values are means ± SD based on three biological replicates. Different lowercase letters indicate significant differences, as determined by Duncan’s multiple range test with P < 0.05.

### 3.4 Differentially expressed genes analysis

**Figure 2:**
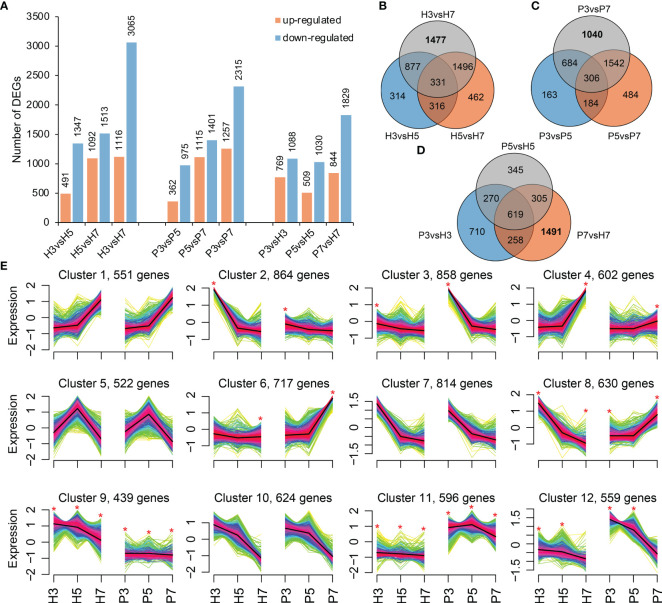
The differentially expressed genes (DEGs) analysis. **(A)** The number of DEGs in pairwise comparison groups. **(B, C)** Venn diagram of DEGs at different developmental stages of Hongfei and Pengzhoubai, respectively. **(D)** Venn diagram of DEGs between Hongfei and Pengzhoubai at three developmental stages. **(E)** Fuzzy cMeans clustering of gene expression trends. Asterisk indicates that the DEGs exhibit different expression patterns between two varieties. A total of 7,776 DEGs were used for the clustering analysis.

The differentially expressed genes were identified by comparisons within intra-varieties at different stages (H3 vs. H5, H5 vs. H7, H3 vs. H7 and P3 vs. P5, P5 vs. P7, P3 vs. P7; comparison I) and inter-varieties at the same stage (P3 vs. H3, P5 vs. H5, and P7 vs. H7; comparison II). In comparison I, a total of 8,624 and 7,425 DEGs were obtained in Hongfei and Pengzhoubai, respectively (**Figure 2A**). The number of down-regulated genes was substantially higher than that of up-regulated genes in both varieties. The most DEGs were observed in H3 vs. H7 and P3 vs. P7 comparison pairs (**Figure 2A**), with 1,477 and 1,040 DEGs being unique in this pair for Hongfei and Pengzhoubai (**Figures 2B, C**). In comparison II, 1,857, 1,539, and 2,673 DEGs were identified at S3, S5, and S7 stage, respectively (**Figure 2A**). 619 common DEGs were identified among three stages, and 1,491 DEGs were unique for S7 stage between two varieties (**Figure 2D**).

To identify the different expression patterns during fruit development between Hongfei and Pengzhoubai, 7,776 DEGs were grouped into clusters using the Fuzzy cMeans clustering method (**Figure 2E**). A total of 12 clusters were generated, among which eight clusters showed obviously different expression pattern between the two varieties, including clusters 2, 3, 4, 6, 8, 9, 11, and 12. Clusters 9 and 11 contained 439 and 596 genes that were differentially expressed at all stages between them, respectively. Genes from clusters 8 and 12 exhibited different expression patterns at S3 and S7 stages, and S3 and S5 stages, respectively. Genes belonging to clusters 2 and 3 showed different expression patterns at S3 stage between them, and genes from clusters 4 and 6 being different at the S7 stage (**Figure 2E**; **Supplementary Figure 1**). These genes in cluster 8 were mainly enriched in “plant hormone signaling”-related pathways by GO enrichment analysis, which exhibited opposite trend between Hongfei and Pengzhoubai with fruit ripening.

GO analysis was employed to classify DEGs annotations between stages and varieties to analyze DEGs’ biological functions. Most of DEGs were enriched in cellular components (CCs), followed by molecular functions (MFs), in which the “catalytic activity” includes the largest number of genes (**Supplementary Figure 2**). Among the cellular components, “cell wall” was enriched for both stages and varieties. Of the biological processes (BPs), “cell wall modification”, “response to hormone”, and “hormone-mediated signaling pathway” were significantly enriched between stages and varieties. Furthermore, “cell wall organization or biogenesis”, “lignin metabolic process”, “lignin catabolic process”, “lignin biosynthetic process”, “pectin catabolic process”, “hemicellulose metabolic process”, and “response to abscisic acid” were significantly enriched during fruit development rather than between varieties. Interestingly, “positive regulation of abscisic acid-activated signaling pathway” and “abscisic acid-activated signaling pathway” were only enriched in H3 vs. H5 comparison group. On the other hand, the most enriched pathways in KEGG analysis were “metabolism”, “transporters”, “biosynthesis of other secondary metabolites”, “phenylpropanoid biosynthesis”, as well as “plant hormone signal transduction” (**Supplementary Figure 3**). Above results suggested that cell wall metabolism pathway and plant hormone signal transduction pathways were closely related to Chinese cherry fruit softening.

### 3.5 DEGs related to cell wall metabolism and hormone signal transduction pathways

#### 3.5.1 Gene expression profiles related to cell wall metabolism

**Figure 3:**
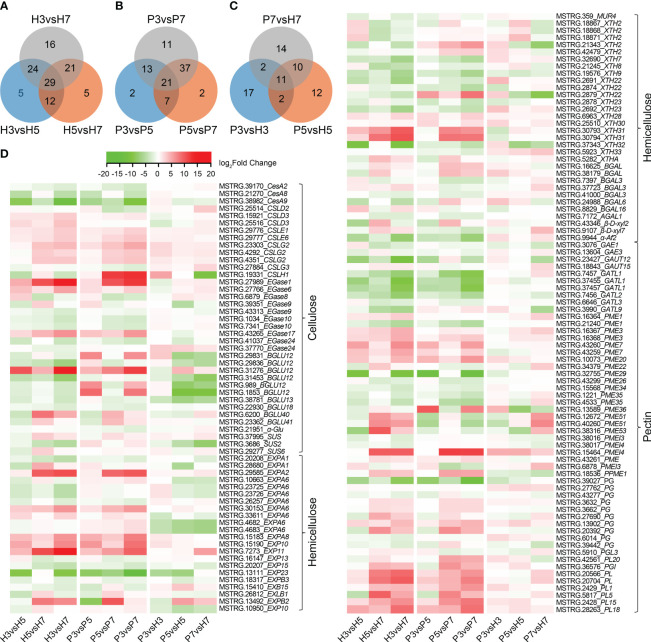
Heatmap display of DEGs involved in cell wall metabolic pathways in Chinese cherry fruits. **(A**, **B)** Venn diagram of DEGs for comparisons between different developmental stages in Hongfei and Pengzhoubai varieties (comparison I). **(C)** Venn diagram of DEGs for comparisons between Hongfei and Pengzhoubai at three developmental stages (comparison II). **(D)** Heatmap of DEGs related to cellulose, hemicellulose, and pectin metabolic pathways. The color scale indicates expression levels as log_2_fold-change values for comparison groups. CesA/CSL, cellulose synthase A/like protein; EGase, endoglucanase; BGLU, β-glucosidase; SUS, sucrose synthase; α-Glu, α-glucosidase; EXP, expansin; MUR, UDP-arabinose 4-epimerase; XTH, xyloglucan endotransglucosylase/hydrolase; BGAL, β-galactosidase; β-D-xyl, β-D-xylosidase; AF, α-L-arabinofuranosidase 2; GAE, UDP-glucuronate 4-epimerase; GAUT, galacturonosyltransferase; PME, pectinesterase; PG, polygalacturonase; PL, pectate lyase.

Analysis of cell wall-related genes identified 112 DEGs and 93 DEGs in Hongfei and Pengzhoubai (comparison I), and 68 DEGs from an inter-variety comparison (comparison II), respectively, whose substrates were cellulose, hemicellulose and pectin (**Figures 3A–C**; **Supplementary Tables 3**–**5**). Based on the expression heatmap (**Figure 3D**), the transcript level of 7 *CSL*, 3 *CEL*, 2 *BGLU* genes in cellulose metabolism increased during fruit softening in Hongfei fruits, especially for *EGase1*, *BGLU12*, and *EGase6*. Other genes, i.e. 3 *CesA*, 4 *EGase*, and 3 *BGLU*, revealed the opposite expression profiles, which were significantly down-regulated with fruit softening. Compared with Pengzhoubai fruits, 2 *CSL* genes were significantly up-regulated, while 5 *BGLU* genes were down-regulated in Hongfei fruits. For the hemicellulose pathway, the expression levels of most *EXP* and *BGAL* genes increased with fruit ripening in both fruits, which were down-regulated in Hongfei fruits through developmental stages. The *XTH* genes showed different expression profiles, i.e. 4 *XTH* genes were significantly increased with fruit ripening, while 8 *XTH* genes were decreased through stages. *XTH2* (MSTRG.21343) and *XTH22* (MSTRG.2879) were dramatically down-regulated in Hongfei fruits at S5 and S7 stages. For pectin pathway, the expression levels of all *PG* and *PL*, and 7~10 *PME* genes were significantly increased, while all *GAUT* and 7~9 *PME* genes were decreased during fruit ripening. The *PME* and *PL* genes were mainly up-regulated in Hongfei fruits, while no significant differences were observed for *PG* genes. These results further confirmed the pivotal role of cellulose, hemicellulose and pectin metabolism for fruit softening.

**Figure 4:**
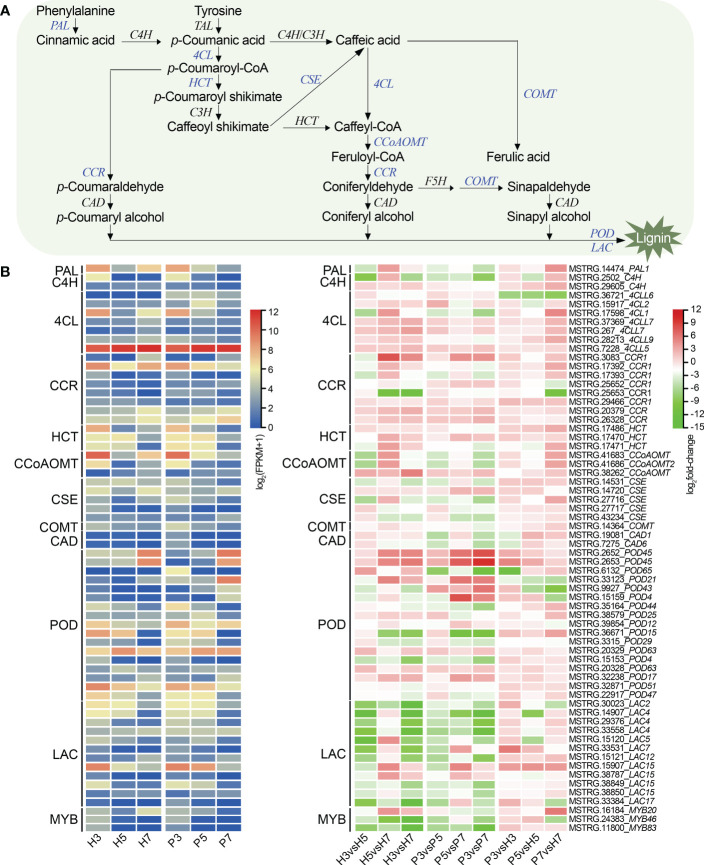
Gene expression profiles in the lignin biosynthesis pathway in Chinese cherry fruits. **(A)** A simplified model of the lignin biosynthesis pathway in Chinese cherry. **(B)** Heatmap representation of DEGs related to lignin biosynthesis. The color scales indicates expression levels as log_2_(FPKM + 1) and log_2_fold-change values, respectively. PAL, phenylalanine ammonium lyase; TAL, tyrosine ammonia-lyase; C4H, cinnamate-4-hydroxylase; 4CL, 4-coumarate-CoA ligase; CCR, cinnamoyl-CoA reductase; HCT, shikimate O-hydroxycinnamoyltransferase; C3H, *p*-Coumarate 3-hydroxylase; CCoAOMT, caffeoyl-CoA O-methyltransferase; F5H, ferulate 5-hydroxylase; CSE, caffeoyl shikimate esterase; COMT, caffeic acid 3-O-methyltransferase; CAD, cinnamyl alcohol dehydrogenase; POD, peroxidase; LAC, laccase.

To investigate the lignin metabolism during fruit development of Chinese cherry, we constructed the heat map based on the expression levels of 64 DEGs (**Figure 4**; **Supplementary Table 6**). Compared with Pengzhoubai, the expression levels of *CCR* and *CCoAOMT* genes were up-regulated at S7 stage in Hongfei fruits. Some homologous genes showed different expression profiles during fruit development. For example, genes encoding *POD* enzymes, *POD21* (MSTRG.33123), *POD45* (MSTRG.2652, MSTRG.2653), and *POD63* (MSTRG.20329) genes, were dramatically increased, but *POD15* (MSTRG.36671), *POD47* (MSTRG.22917), and *POD51* (MSTRG.32871) genes decreased with fruit ripening. Most of *POD* genes were up-regulated in Hongfei fruits at three stages. All the *LAC* genes were significantly decreased with fruit ripening. Our results suggested that *CCR*, *POD*, and *CCoAOMT* were involved in the regulation of lignin biosynthesis in Chinese cherry. However, *C4H*, *CAD*, *COMT*, and *F5H* showed no significant differences between varieties. A transcription factor *MYB20* (MSTRG.16184), homologous to *MYB20* from *Arabidopsis*, was up-regulated in Hongfei fruits at S7 stage (**Figure 4**), which was shown to positively regulate lignin biosynthesis during secondary cell wall formation (Geng et al., 2020). *CpMYB46* (MSTRG.24383) and *CpMYB83* (MSTRG.11800), two lignin-specific TFs in *Arabidosis* (Xiao et al., 2021), directly modulate the lignin synthesis, which were decreased sharply during fruit ripening (**Figure 4**).

#### 3.5.2 Gene expression profiles related to plant hormone signaling

**Figure 5:**
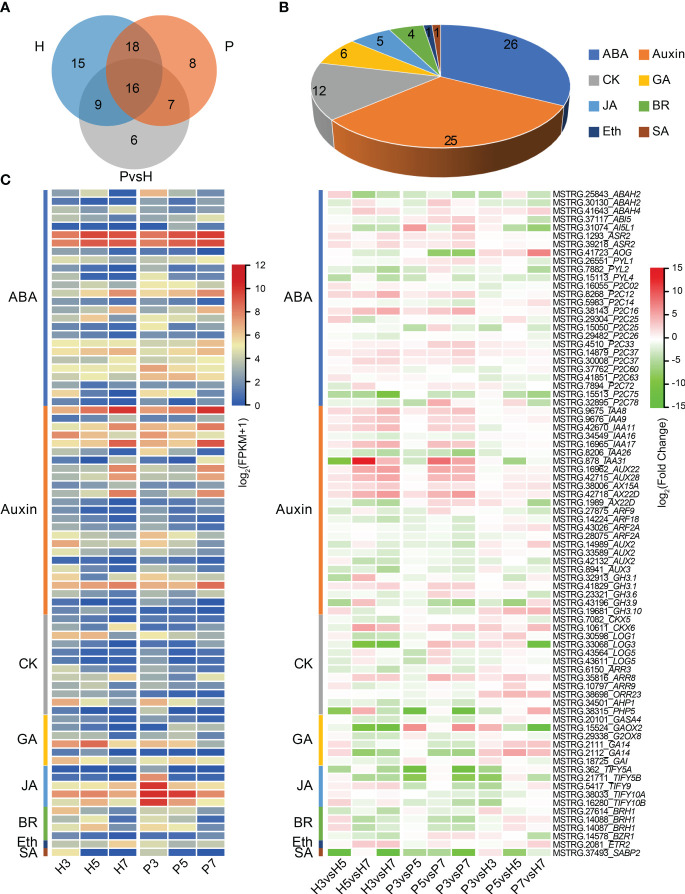
Differential expression of plant hormone signaling genes in Chinese cherry fruits. **(A)** Venn diagram representing the extent of overlap between genes identified in the indicated pairwise comparisons. H and P refer to DEGs in comparisons between different developmental stages in Hongfei and Pengzhoubai (comparison I). P vs. H refers to DEGs in comparisons between the two varieties in three developmental stages (comparison II). **(B)** Number of differentially expressed plant hormone signaling genes. **(C)** Heatmap representation of DEGs related to different plant hormones. The color scales indicate expression levels as log_2_(FPKM + 1) and log_2_fold-change values, respectively. ABA, abscisic acid; CK, cytokinin; GA, gibberellic acid; JA, jasmonic acid; BR, brassinosteroid; Eth, ethylene; SA, salicylic acid.

To explore the role of plant hormones in fruit ripening and softening of Chinese cherry, we further identified the DEGs associated with plant hormone signaling. The result showed that 80 genes were associated with eight phytohormones (**Figure 5**; **Supplementary Table 7**). 58, 49 and 38 DEGs were identified in Hongfei and Pengzhoubai (comparison I), and inter-varieties comparison (comparison II), respectively (**Figure 5A**). The DEGs related to abscisic acid and auxin accounted for more than half of the total phytohormones (**Figure 5B**). Based on the expression heatmap (**Figure 5C**), most of ABA related genes were dramatically decreased with fruit ripening in the two varieties, while several genes were increased especially *CpP2C12* and *CpP2C16*. Most of them were down-regulated in Hongfei fruits compared with Pengzhoubai, except for *AOG* and ABAH4 genes. Auxin related genes including IAA and AUX were mainly increased during fruit development, especially *IAA8*, *IAA9*, *AUX22*, and *AUX28*, whereas no obvious differences were observed between two varieties. The expression levels of four *ARFs* were significantly declined with fruit ripening, and *ARF9* was down-regulated in Hongfei fruits. Most GA, JA, BR, and SA genes were down-regulated with fruit ripening for both two varieties. The JA related genes were all down-regulated in Hongfei fruits over the same stage. Only one gene, *ETR2*, identified from DEGs, was decreased in Hongfei, while no obvious change was detected in Pengzhoubai with fruit ripening. These results suggested that plant hormone signaling genes played important roles with fruit ripening of Chinese cherry, especially ABA and auxin.

#### 3.5.3 Gene expression profiles of transcription factors

**Figure 6:**
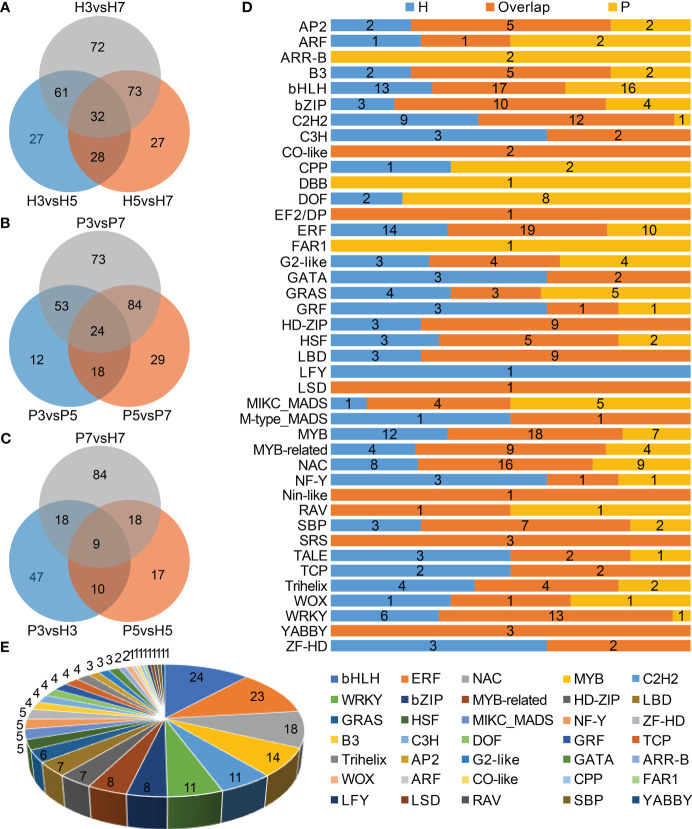
Characterization of differentially expressed transcription factor (TF) genes in Chinese cherry fruits. **(A, B)** Venn diagrams of differentially expressed TF genes in different developmental stages in Hongfei and Pengzhoubai fruits (comparison I). **(C)** Venn diagram of differentially expressed TF genes in comparisons between the two varieties (comparison II). **(D)** Distribution of differentially expressed TF families in comparisons between different developmental stages in Hongfei and Pengzhoubai. **(E)** Number of differentially expressed TF genes identified in comparisons between the two varieties.

**Figure 7:**
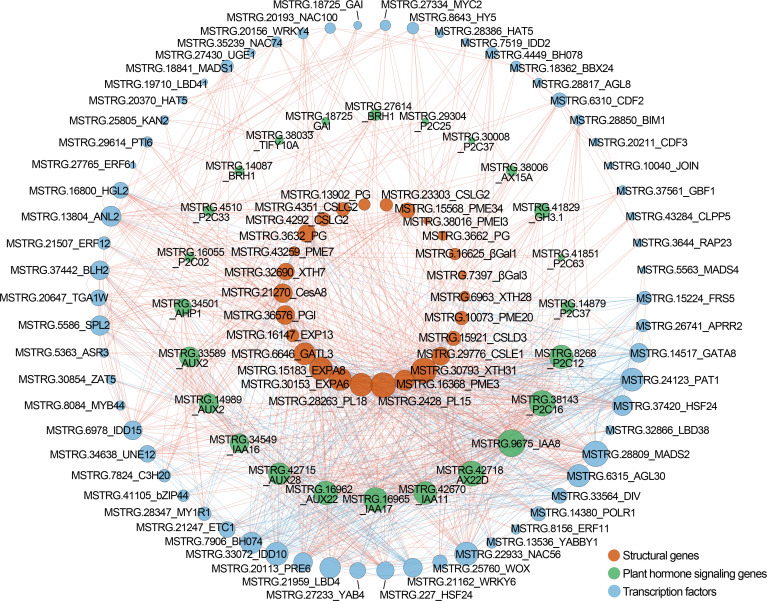
Correlation network among structural genes, plant hormone signaling genes, and TF genes in Chinese cherry fruits. The red, green, and blue circles represent structural genes, plant hormone signaling genes, and TFs, respectively. The red and blue lines represent the positive and negative correlation. The gene descriptions are listed in **Supplementary Table 9**.

In total, 447 DEGs were identified as transcription factors belonging to 41 families, which contained bHLH, ERF, MYB, NAC, C2H2, and WRKY, etc. (**Supplementary Table 8**). The top three families of Chinese cherry TFs with largest number of genes were bHLH (*n*=50), ERF (*n*=44), and MYB (*n*=40). We further identified 320 and 293 TF genes belonging to 31 and 27 families in Hongfei and Pengzhoubai respectively (**Figures 6A, B, D**). 203 TF genes belonging to 35 families were screened between the two varieties (**Figures 6C, E**). Next, the correlation network analysis revealed that 26 key structural genes containing *CSL*, *PG*, *PL*, *PME*, *XTH*, and *EXP* are correlated with 24 phytohormone-related genes and 62 TF genes (**Figure 7**; **Supplementary Table 9**). The heatmap based on expression levels showed that most TFs were down-regulated with fruit ripening, except for bHLH, LBD, HD-ZIP, and several MADS, MYB, and NAC genes (**Supplementary Figure 6**). There were no significant differences for most TFs at three stages between the two varieties. Several TFs such as C3H20, JOIN, MYB44, and NAC100 were significantly down-regulated at S7 stage in Hongfei fruits. The results indicated that the phytohormone-mediated transcriptional network played a crucial role in fruit softening and ripening of Chinese cherry.

### 3.6 Weighted gene co-expression network association analysis

**Figure 8:**
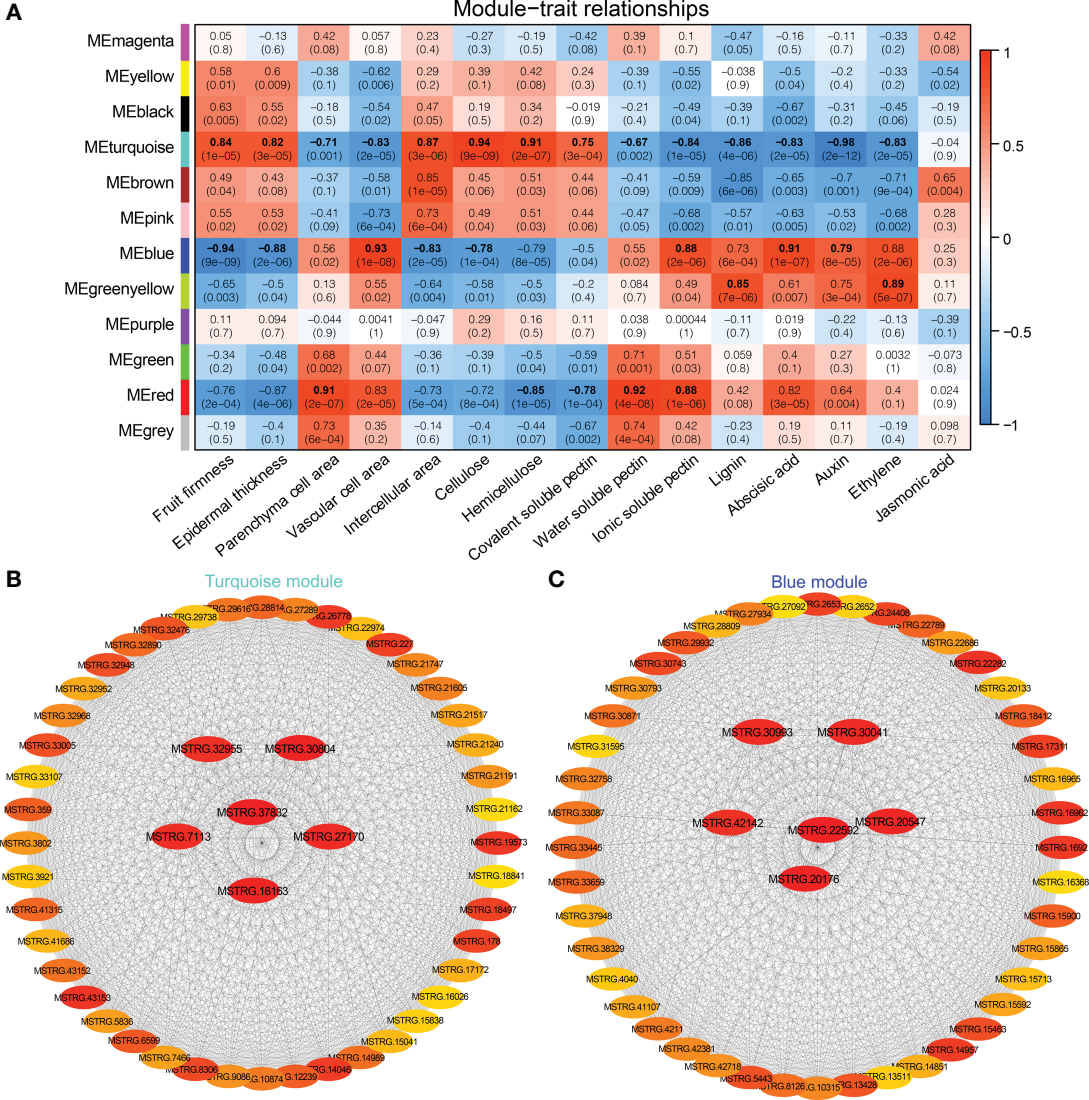
WGCNA of DEGs identified from different stages and varieties in Chinese cherry fruits. **(A)** Module-trait relationships. The colors from blue to red represent *r^2^* values from −1 to 1. **(B, C)** Gene co-expression networks and hub genes in module Turquoise and Blue. The gene description is given in **Supplementary Tables 10**, **11**, respectively.

The WGCNA was carried out based on the normalized expression data for 7,776 DEGs from 18 samples. After filtering, we retained 1,944 genes that were divided into 12 distinct gene models (**Supplementary Figure 4**). Based on Pearson correlation analysis, the relationship was quantified between each module and phenotype to further generate a heat map (**Figure 8A**). Among them, fruit firmness, epidermal thickness, intercellular area, and cellulose were significantly associated with the Turquoise and Blue modules, and the correlation coefficients (*r^2^*) ranged from 0.82 to 0.94, and from −0.94 to −0.78, respectively. Hemicellulose and covalent-soluble pectin were positively correlated with the Turquoise module (*r^2^ = *0.91, 0.75), and negatively correlated with the Red module (*r^2^* = −0.85, −0.78). Parenchyma cell area, water-soluble pectin and ionic-soluble pectin were significantly correlated with the Red and Turquoise modules, and the correlation coefficients (*r^2^*) was 0.91, 0.92, 0.88, and −0.71, −0.67, −0.84, respectively. Vascular cell area, and ABA, IAA contents were significantly positively correlated with the Blue module (*r^2^ = *0.79 ~ 0.93), and negatively correlated with the Turquoise module (*r^2^* = −0.98 ~ −0.83). Both lignin and JA contents were positively correlated with Greenyellow module, and negatively correlated with Turquoise module. Turquoise, Blue, Red and Greenyellow modules harbored 645, 365, 94, and 47 DEGs, respectively (**Supplementary Tables 10**–**13**).

We then applied function enrichment analysis of genes from Turquoise and Blue modules. GO enrichment analysis demonstrated that the Turquoise module genes were mainly enriched in “cell wall organization or biogenesis”, “cell wall organization”, “polysaccharide metabolic process”, “pectinesterase activity” and other processes, and 9, 9, 7, and 6 genes related to “cell wall polysaccharide metabolic process”, “pectin metabolic process”, “hemicellulose metabolic process”, and “lignin metabolic process” were screened (**Supplementary Figure 5**). The Blue module genes were mainly enriched in “cell wall organization or biogenesis”, “hormone-mediated signaling pathway”, “cell wall organization”, “response to abscisic acid”, “cell wall”, etc. Then, KEGG enrichment analysis showed that most of Turquoise module genes participated in the “energy metabolism”, “photosynthesis proteins”, “metabolism” and “fructose and mannose metabolism” pathways, and the genes of Blue module were mainly enriched in the “metabolism”, “signal transduction”, and “plant hormone signal transduction” pathways (**Supplementary Figure 5**).

The connectivity value of the genes in Turquoise and Blue modules were calculated to screen the hub genes related to fruit softening, and the interaction network was exported by CytoHubba analysis (**Figures 8B, C**). The hub genes in Turquoise module were MSTRG.37832, MSTRG.30804, and MSTRG.16163. MSTRG.37832 and MSTRG.16163 encoded protein MID1-COMPLEMENTING ACTIVITY 1 and TPX2 and were linked to 472 and 419 genes, respectively (**Supplementary Table 10**). MSTRG.30804 encoded a *F8H* gene and was linked to 437 genes, which was involved in the synthesis of hemicellulose glucuronoxylan (Lee et al., 2009). MSTRG.22592, MSTRG.20176, and MSTRG.20547 were selected as the hub genes in the Blue module (**Supplementary Table 11**). MSTRG.22592 encoded serine/threonine-protein kinase *WAG1*, linked to 261 genes, which was involved in the regulation of auxin signaling (Dhonukshe et al., 2010). These findings suggested that these genes were significantly correlated with the fruit softening of Chinese cherry.

### 3.7 Verification of DEGs by RT-qPCR

**Figure 9:**
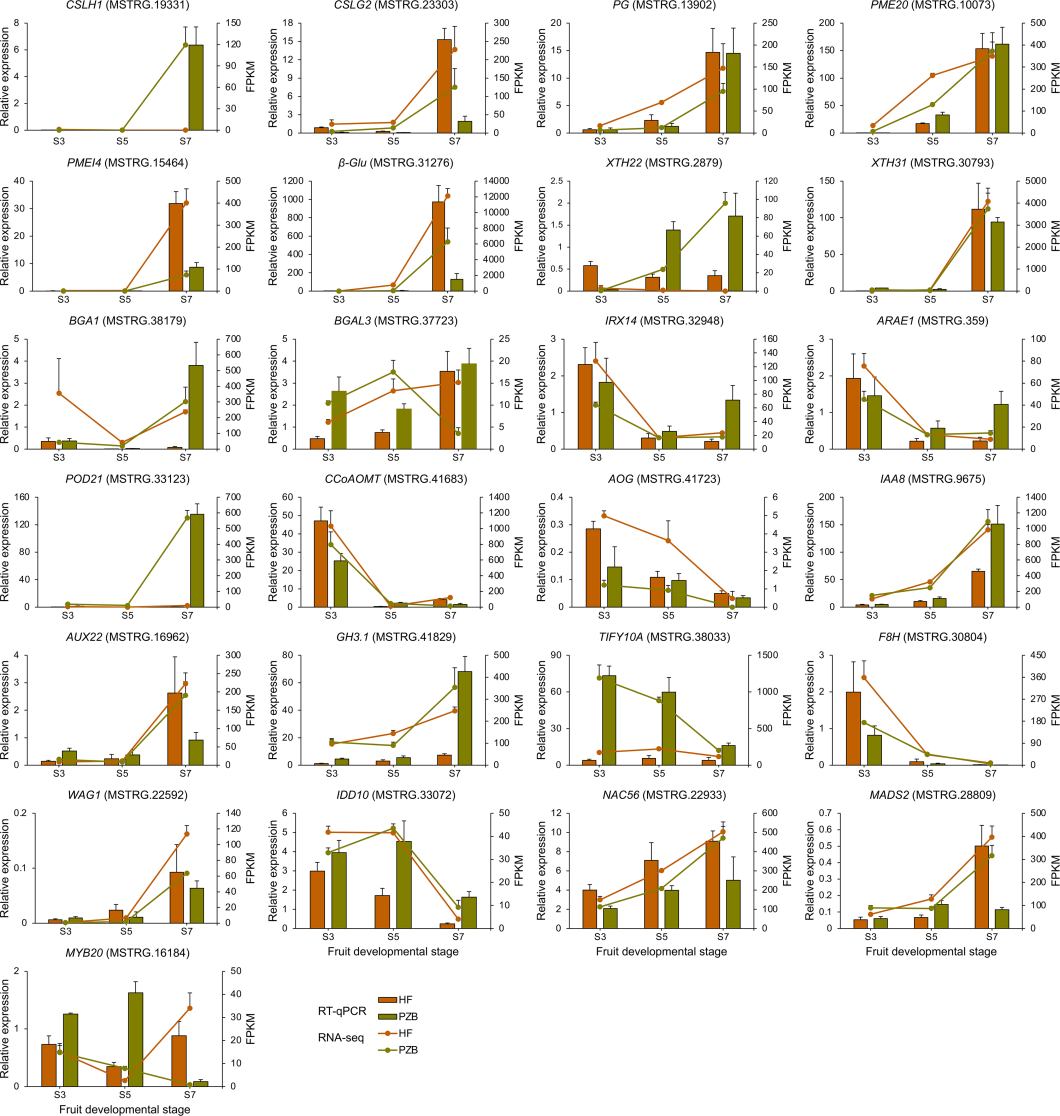
Validation of RNA-seq data by RT-qPCR. Relative expression level determined by RT-qPCR and expressed in 2^−△△Ct^ is shown by the histogram. Transcript level obtained by RNA-seq is shown as FPKM values by the line chart. Data are shown as means ± SD of three independent biological replicates.

Twenty-five DEGs related to cell wall metabolism, plant hormone signaling, and TF genes were selected for verification (**Figure 9**). The expression patterns from the two varieties were largely consistent in the expression trends for the 25 genes between the RT-qPCR and RNA-seq data. For the 12 genes involved in cell wall metabolism, ten genes are positively correlated with fruit ripening, whose expression levels increased during fruit development especially after S5 stage. *CpCSLH1*, *CpPME20*, *CpXTH22*, and *CpBAG1* showed significantly higher expression levels in Pengzhoubai than that in Hongfei fruits, indicating that the degradation rate of cell wall is faster in Pengzhoubai. The expression levels of *CpIRX14* and *CpARAE1* decreased sharply across conversion period, then slightly increased to S7 stage. Both of them showed higher expression levels at S3 stage, but lower levels from S5 stage in Hongfei fruits compared with Pengzhoubai. For *CpCCoAOMT* gene involved in lignin biosynthesis, its expression dramatically decreased with fruit ripening, but up-regulated through development in Hongfei fruits. The expression of *CpTIF10A* kept at stable level during fruit ripening in Hongfei, but it showed much higher levels through fruit development in Pengzhoubai. For TF genes, *CpNAC56* and *CpMADS2* exhibited similar expression trends with fruit ripening, while *CpMYB20* showed different expression patterns between the two varieties. Thus, the expression patterns of these selected genes examined in the RNA-seq analysis were accordant with the RT-qPCR results.

## 4 Discussion

### 4.1 Cell wall degradation with fruit softening of Chinese cherry

Fruit softening within fruit ripening process is involved in extensive changes including physiology, biochemistry, cell wall structure and composition, and gene expression (Shi et al., 2022; Zhai et al., 2022). The ultrastructure and metabolism of cell wall have been previously reported to be key factors during the softening of fleshy fruits (Vicente et al., 2007; Wang et al., 2019). The physiological basis associated with fruit softening mainly attribute to the modification or breakdown of cell wall polysaccharides (Brummell et al., 2004; Zhai et al., 2022). Recent studies show that the difference in fruit firmness between varieties was determined by the different degradation rate of cell wall materials. Hard-fleshed varieties contained higher contents of cellulose, hemicellulose, and pectin than soft-fleshed varieties in apples (Yang et al., 2018) and blueberries (Liu et al., 2019). In sweet cherry, the degradation rate of hemicellulose and pectin determined the different firmness between hard-fleshed and soft-fleshed varieties (Zhai et al., 2022). Our results were in part consistent with the discovery in sweet cherry. Transcriptomic analysis revealed that the structural genes were mainly participated in cellulose, hemicellulose and pectin metabolism in the comparison between Hongfei and Pengzhoubai (**Figures 3C, D**). Cell wall thickness and strength are also key contributors to fruit firmness (Saladié et al., 2007). The changes in cell components and structure, particularly those directly impacting cell wall thickness and strength, affect the textural changes during fruit ripening process. In this study, larger epidermal thickness, and smaller and tighter mesocarp cells were observed in Hongfei compared with Pengzhoubai fruit, consistent with the findings in hard pericarp of passion fruit (Tian et al., 2022). Thus, our results indicated that the cell wall metabolism exhibited distinct contribution to fruit softening in Chinese cherry.

Cell wall metabolism is an important event during fruit ripening, and the disintegrating of multiple polysaccharide networks was performed by various families of cell wall-degrading enzymes. Cellulose, hemicellulose, and pectin are the major classes of cell wall polysaccharides that involve dramatical changes during ripening (Prasanna et al., 2007; Li et al., 2010). Celluloses are very stable structures, which are essential in maintaining fruit firmness (Olmedo et al., 2021; Su et al., 2022). Genes related to cellulose, hemicellulose, and pectin metabolism exhibited the similar expression patterns, and most of them were significantly increased during fruit ripening (**Figure 3D**; **Supplementary Tables 3**, **4**). This was consistent with those findings for other fruits, such as sweet cherry (Zhai et al., 2022) and papaya (Zhu et al., 2019). Compared with Pengzhoubai fruits, the expression levels of genes involved in cellulose and hemicellulose metabolism were down-regulated, while genes involved in pectin metabolism were mainly up-regulated in Hongfei fruits, especially at S5 and S7 stages (**Supplementary Table 5**). These results coincided with the differences of cell wall components between the two varieties (**Table 1**). Thus, our findings suggested that cellulose and hemicellulose largely contributed to maintain the fruit firmness of Chinese cherry.

Expansin can bind to glucan-coated cellulose to break the chemical bond between microfibrils and glucans, leading to fruit cell wall loosening (Cosgrove, 2015). EXP genes take place during fruit softening, which are characterized with different expression profiles in different fleshy fruits. In Chinese cherry, the expression levels of most EXPs increased significantly during the softening (**Figure 3**; **Supplementary Tables 3**, **4**), which was accordant with that in sour cherry (Yoo et al., 2003) and strawberry fruits (Valenzuela-Riffo and Morales-Quintana, 2020; Valenzuela-Riffo et al., 2020). These genes were all down-regulated in Hongfei compared with Pengzhoubai fruits (**Figure 3**; **Supplementary Table 5**), consistent with the microstructure difference between them (**Figures 1C, D**). The above results might indicate a relative low level of cell wall loosening with fruit ripening in Hongfei fruits.

Lignin is an essential compound, functioning as the structural support of the cell walls and response to various abiotic and biotic stresses (Liu et al., 2018). The expression levels of *POD*, *PAL*, *C4H*, and *4CL* were positively correlated with lignin content in loquat fruit (Cai et al., 2006). The lignin content in papaya fruits slightly decreased with fruit ripening, and the gene families involved in lignin metabolism showed different expression profiles (Zhu et al., 2019). In the present work, majority of these genes were decreased with fruit softening, but they were mainly up-regulated in Hongfei fruits, especially at S7 stage (**Figure 4**). Hongfei fruits contain more lignin content than that of Pengzhoubai at ripening stage (**Table 1**) so that it could maintain higher fruit firmness.

### 4.2 Plant hormones involved in fruit softening of Chinese cherry

Plant hormones play important roles in fruit softening and ripening, and this process is more linked to ABA and auxin for non-climacteric fruits, such as sweet cherry (Fresno & Munne-Bosch, 2021), strawberry (Li et al., 2022), and grape (Jia et al., 2017). Endogenous ABA has been confirmed with a pivotal role in the triggering of fruit ripening in sweet cherry, *via* the modulation of ripening-related metabolism pathways such as anthocyanin accumulation (Luo et al., 2014; Wang et al., 2015). ABA positively regulates the development, ripening, and quality through the interaction of *PacPP2C1* with six *PacSnRK2s* (Tijero et al., 2016; Shen et al., 2017). In Chinese cherry, ABA and auxin were established to participate in fruit softening by co-expression network analysis (**Figure 7**). Some ABA related genes were dramatically decreased with fruit ripening in both two varieties, especially *P2C75* (**Figure 5**). The ABA level increased from the green stage through the red stage, but it was significantly higher in Pengzhoubai fruits than Hongfei fruits at S7 stage (**Table 2**). By comparison, the expression levels of ABA receptor genes were significantly down-regulated in Hongfei fruits at S7 stage. This might make Hongfei fruits maintain higher firmness than Pengzhoubai at mature stage.

IAA can inhibit DNA demethylase genes to maintain high methylation levels in the fruit, playing an inhibitory role in fruit ripening (Catalá et al., 2000). The application of exogenous IAA in grapes suppresses the ABA accumulation and diminishes the transcripts of ripening-related genes (*PE*, *PG*, *PL* and *CELL*), delaying fruit softening and ripening (Jia et al., 2017). In sweet cherry, IAA treatment induces the accumulation of ABA, but it delayed the initiation of fruit ripening (Wang et al., 2015). By contrast, NAA (auxin 1-naphthaleneacetic acid) treatment alters ethylene concentration, which stimulates fruit ripening and promotes anthocyanin accumulation through ABA metabolism (Clayton-Cuch et al., 2021). In peach, the overexpression of *PpSAUR43*, a IAA-responsive gene, down-regulates the cell wall modification gene *PpPG* and delays the fruit softening (Wang et al., 2022a). However, Ponce et al. (2021) proposed that IAA negative role is to counteract the ABA effect, preventing the excessive ABA signal amplification, rather than directly related to a delay in sweet cherry. Here, genes involved in auxin synthesis, transport, and signal transduction were identified during fruit softening (**Figure 5**; **Supplementary Table 7**). In addition, JA treatment promoted fruit softening by increasing the expression of *FaMYC2* and *FaJAZ1* in strawberry (Delgado et al., 2018). Here, *CpTIFY10A*, a JA-related gene, showed high expression level through fruit development in Pengzhoubai, which might be involved in Chinese cherry fruit ripening.

### 4.3 TFs involved in fruit softening of Chinese cherry

Fruit softening is also regulated by diverse transcription factors. MADS and NAC genes were thought to be important regulators of softening in non-climacteric fruits including sweet cherry (Qi et al., 2020) and strawberry (Seymour et al., 2011; Martín-Pizarro et al., 2021). *PaMADS7* in sweet cherry can enhance the expression of *PaPG1 via* directly binding to its promoter (Qi et al., 2020). *FaRIF*, encoding *FaNAC035*, is essential to positively regulate strawberry ripening by controlling cell wall degradation process (Martín-Pizarro et al., 2021). Previous report also found that ERF, WRKY, MYB and bHLH were significantly activated with fruit ripening (Kou et al., 2021). AP2/ERF is not only involved in fruit softening but also can regulate the biosynthesis of phytohormones. *MaERF11* binds to promoters of ripening-related genes *MaEXP2/7/8 via* the GCC-box motif, which inhibits banana fruit softening (Han et al., 2016b). *FaWRKY71* facilitate strawberry fruit softening and ripening by promoting the expression of *FaARF6/8* and *FaPG19/21* (Yue et al., 2022). The overexpression of *FvMYB79* dramatically promote the transcription of *FvPME38*, leading to fruit softening in strawberry (Cai et al., 2021). *FaSPT*, a *SPATULA* gene encoding a bHLH protein, is an essential TF involved in strawberry fruit ripening (Tisza et al., 2010).

Our co-expressed network analysis confirmed that TFs including MADS, bHLH, MYB, ERF, NAC, and WRKY were correlated with structural genes related to cell wall metabolism (**Figure 7**). Six *CpMADS* genes were identified (**Supplementary Table 11**), among which *CpMADS2* was up-regulated with fruit ripening (**Supplementary Figure 6**). The expression levels of five bHLH genes were also increased with fruit development, especially for *CpPRE6*. *CpNAC56* were up-regulated with fruit ripening in two varieties, but *CpNAC100* showed opposite expression trends between them (**Supplementary Figure 6**). The expression levels of *CpERF11* was also increased with fruit ripening. Overall, these results supported that these transcription factors also participated in the fruit ripening and softing of Chinese cherry.

## 5 Conclusion

**Figure 10:**
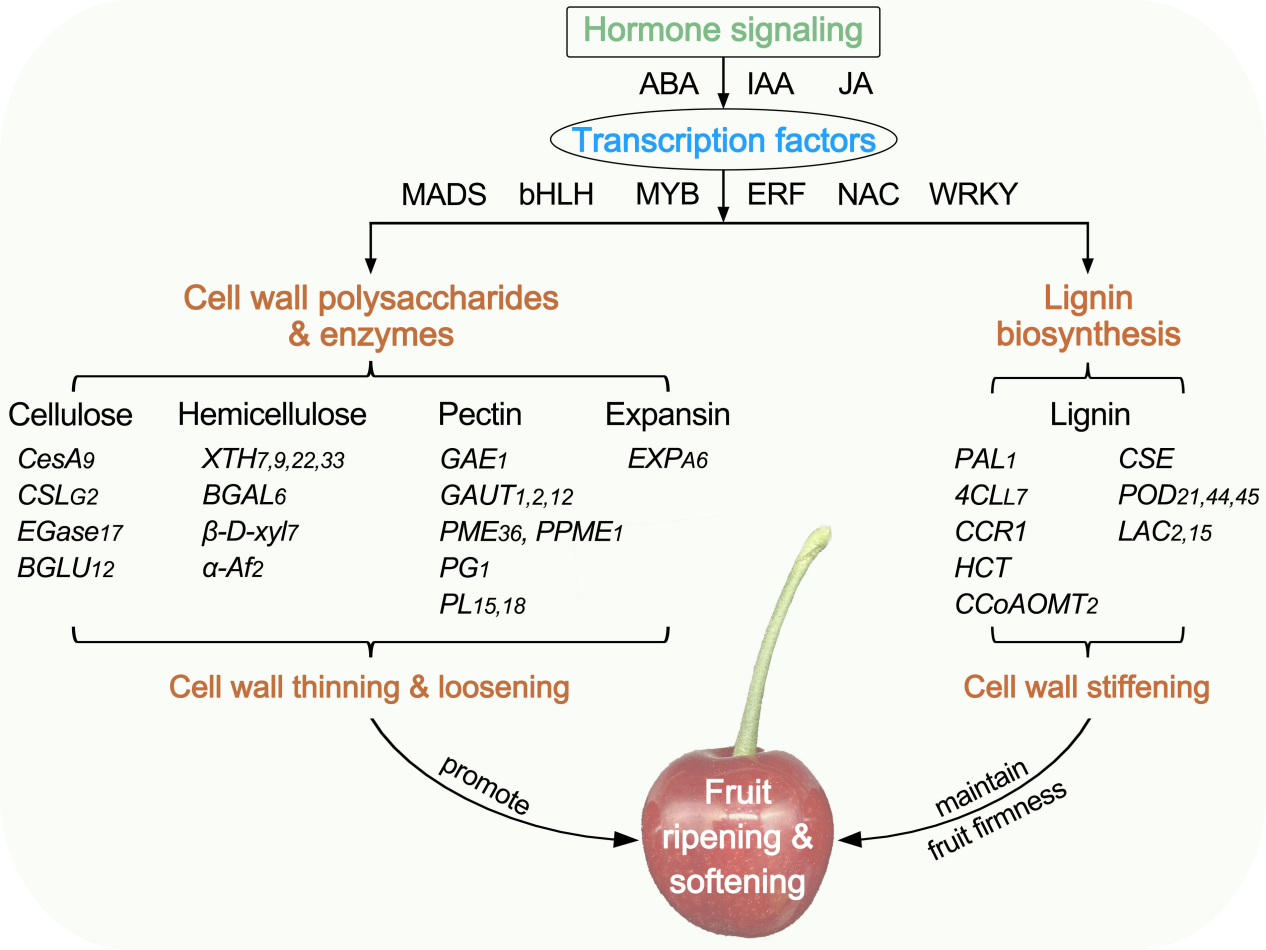
Diagram summarizing the plant cell wall in fruit ripening and softening of Chinese cherry. CesA/CSL, cellulose synthase A/like protein; EGase, endoglucanse; BGLU, β-glucosidase; XTH, xyloglucan endotransglucosylase/hydrolase; BGAL, β-galactosidase; β-D-xyl, β-D-xylosidase; α-Af, α-L-arabinofuranosidase; GAE, UDP-glucuronate 4-epimerase; GAUT, galacturonosyltransferase; PME, pectinesterase; PG, polygalacturonase; PL, pectate lyase; EXP, expansin; PAL, phenylalanine ammonia-lyase; 4CL, 4-coumarate-CoA ligase; CCR, cinnamoyl-CoA reductase; HCT, shikimate O-hydroxycinnamoyltransferase; CCoAOMT, caffeoyl-CoA O-methyltransferase; CSE, caffeoylshikimate esterase, POD, peroxidase; LAC, laccase.

In this study, comparative physiological and transcriptome analyses were conducted to illustrate the differences in fruit softening between hard-fleshed Hongfei and soft-fleshed Pengzhoubai of Chinese cherry. A summary of the cell wall change with fruit ripening and softening is shown in **Figure 10**. The dynamic changes of cellulose, hemicellulose, and pectin contents, the degrading enzyme activities, and the microstructure were closely related to the fruit firmness during fruit softening. The DEGs identified from RNA-seq were mainly enriched the cell wall metabolism and hormone signal transduction pathways. The structural genes exhibited the similar expression patterns with fruit ripening in both two varieties, but mainly down-regulated for cellulose and hemicellulose genes, and up-regulated for lignin biosynthesis pathway genes. These differences might delay the cell wall thinning and loosening, and enhance the cell wall stiffening, which maintained a higher level of fruit firmness in Hongfei fruits than Pengzhoubai. Plant hormone signal genes (ABA, IAA, and JA) and TFs (MADS, bHLH, MYB, ERF, NAC, and WRKY) were involved in fruit softening, supported by co-expressed network analysis.

## Data availability statement

The datasets presented in this study can be found in online repositories. The names of the repository/repositories and accession number(s) can be found in the article/**Supplementary Material**.

## Author contributions

Conceptualization: YW and XW. Methodology: YW and LM. Software: YW, LM, and YM. Validation: LM, JZ and HW. Formal analysis: YW and LM. Investigation: YM, TT, ZL, QC and WH. Resources: YXL, YTZ and ML. Data curation: YZ and YL. Writing—original draft: YW. Writing—review & editing: YW and XW. Supervision: HT. Project administration: SY. Funding acquisition: YW and XW. All authors contributed to the article and approved the submitted version.
