# Supplementary material for: Comparative physiological and transcriptomic analyses provide insights into fruit softening in Chinese cherry [Cerasus pseudocerasus (Lindl.) G.Don]
Source: Front Plant Sci. 2023 Jul 17;14:1190061. doi: 10.3389/fpls.2023.1190061 (PMC10388103; doi:10.3389/fpls.2023.1190061)
Supplement: Supplementary file 1 [file DataSheet_1.zip › Supplementary Figures.DOCX]

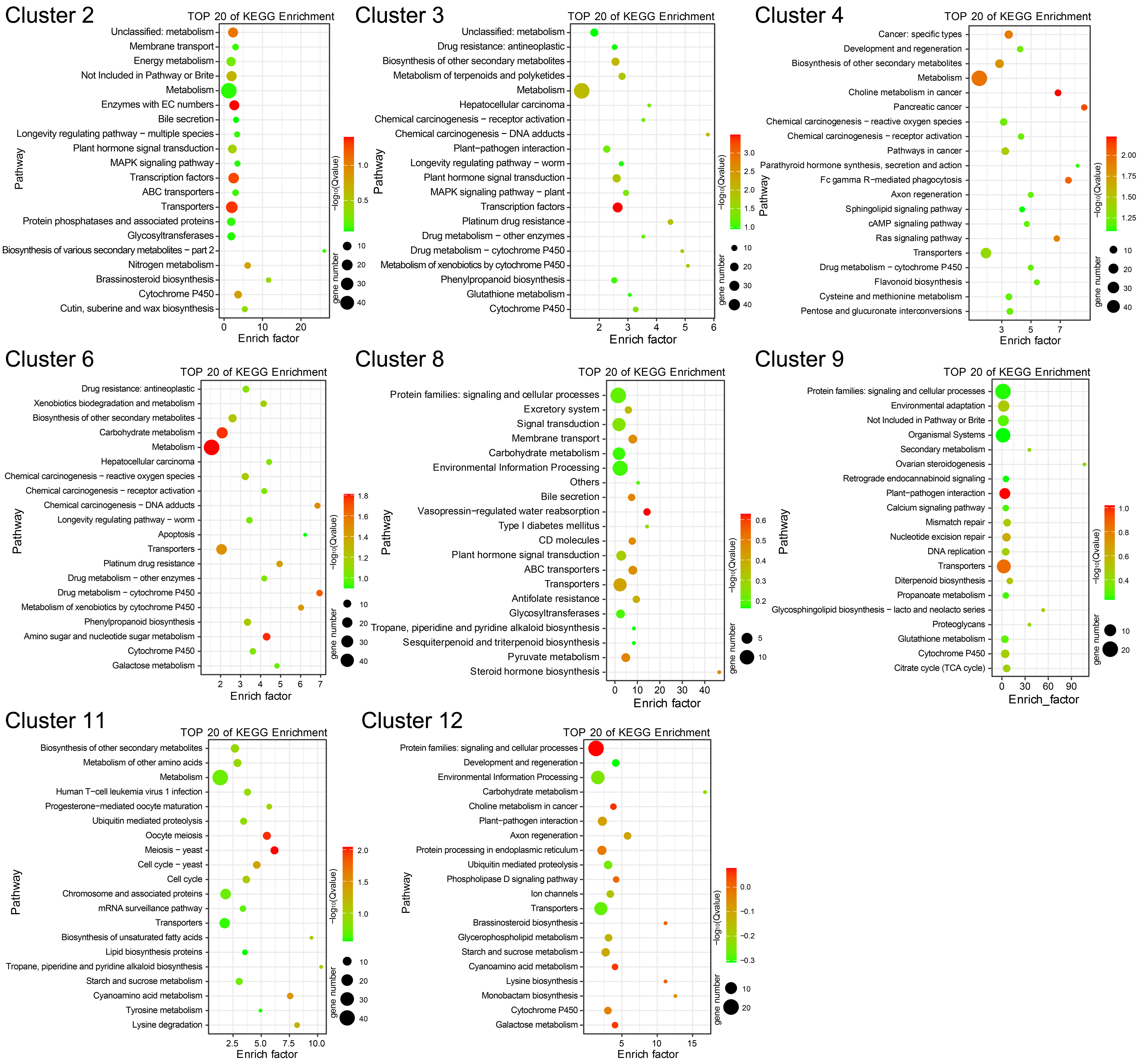


**Supplementary Figure 1** KEGG enrichment analysis of DEGs belonging to 12 clusters.


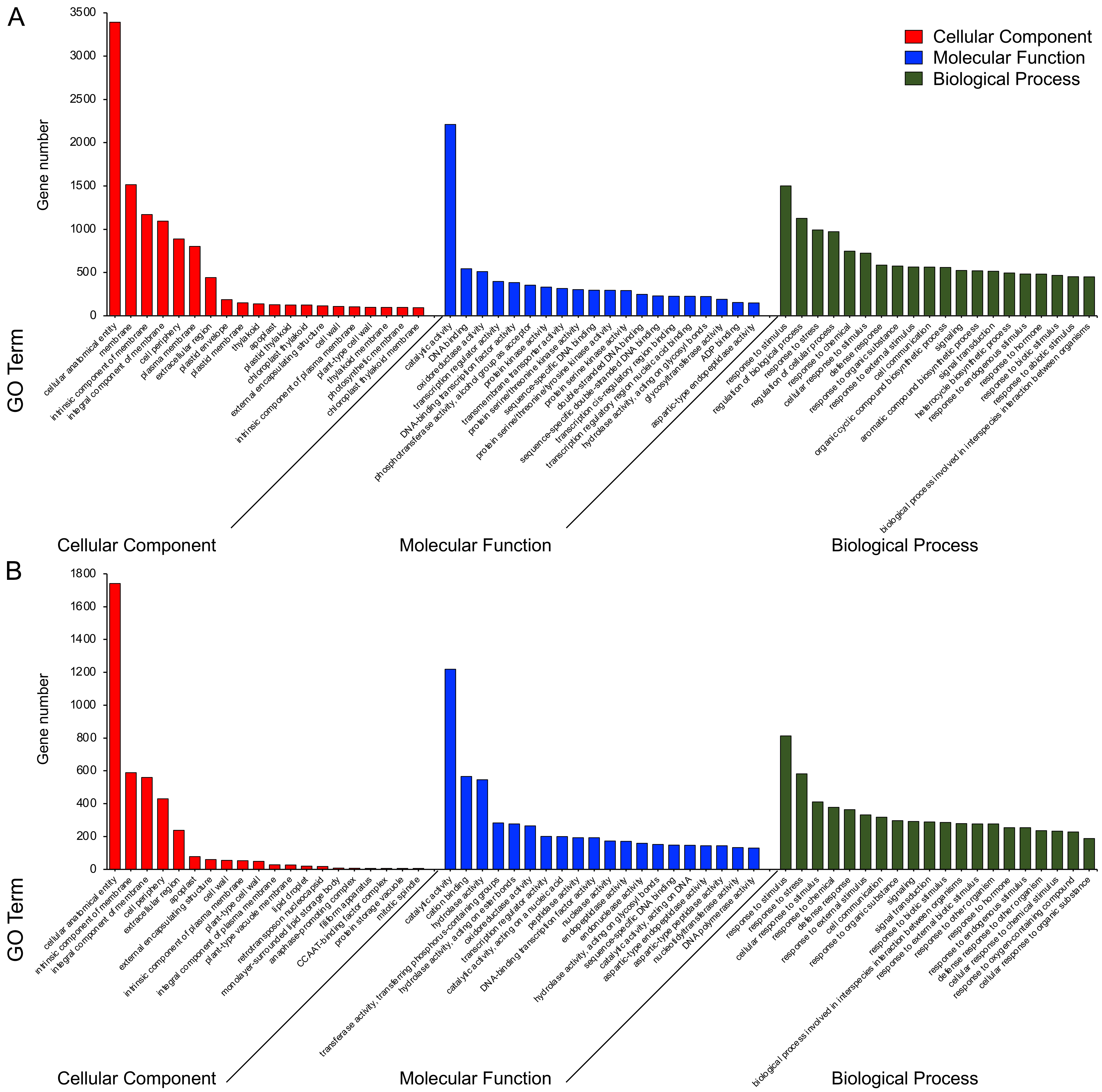


**Supplementary Figure 2** GO enrichment of DEGs among different combinations of stages and varieties. (**A**) A total of 6,757 DEGs of the same variety at different developmental stages. (**B**) A total of 3,998 DEGs of different varieties at the same developmental stage.


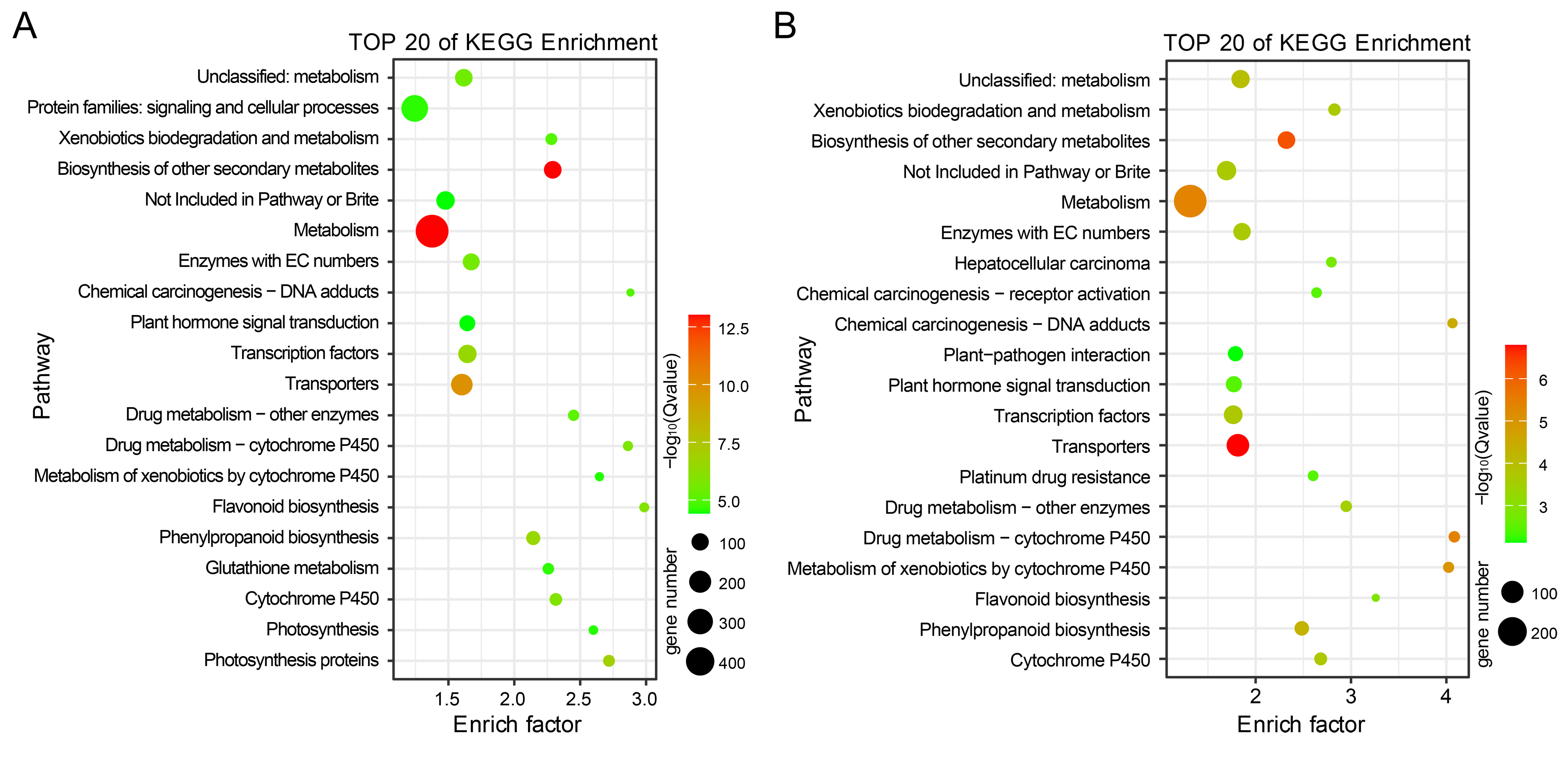


**Supplementary Figure 3** KEGG enrichment of DEGs among different combinations of stages and varieties. (**A**) A total of 6,757 DEGs of the same variety at different developmental stages. (**B**) A total of 3,998 DEGs of different varieties at the same developmental stage.


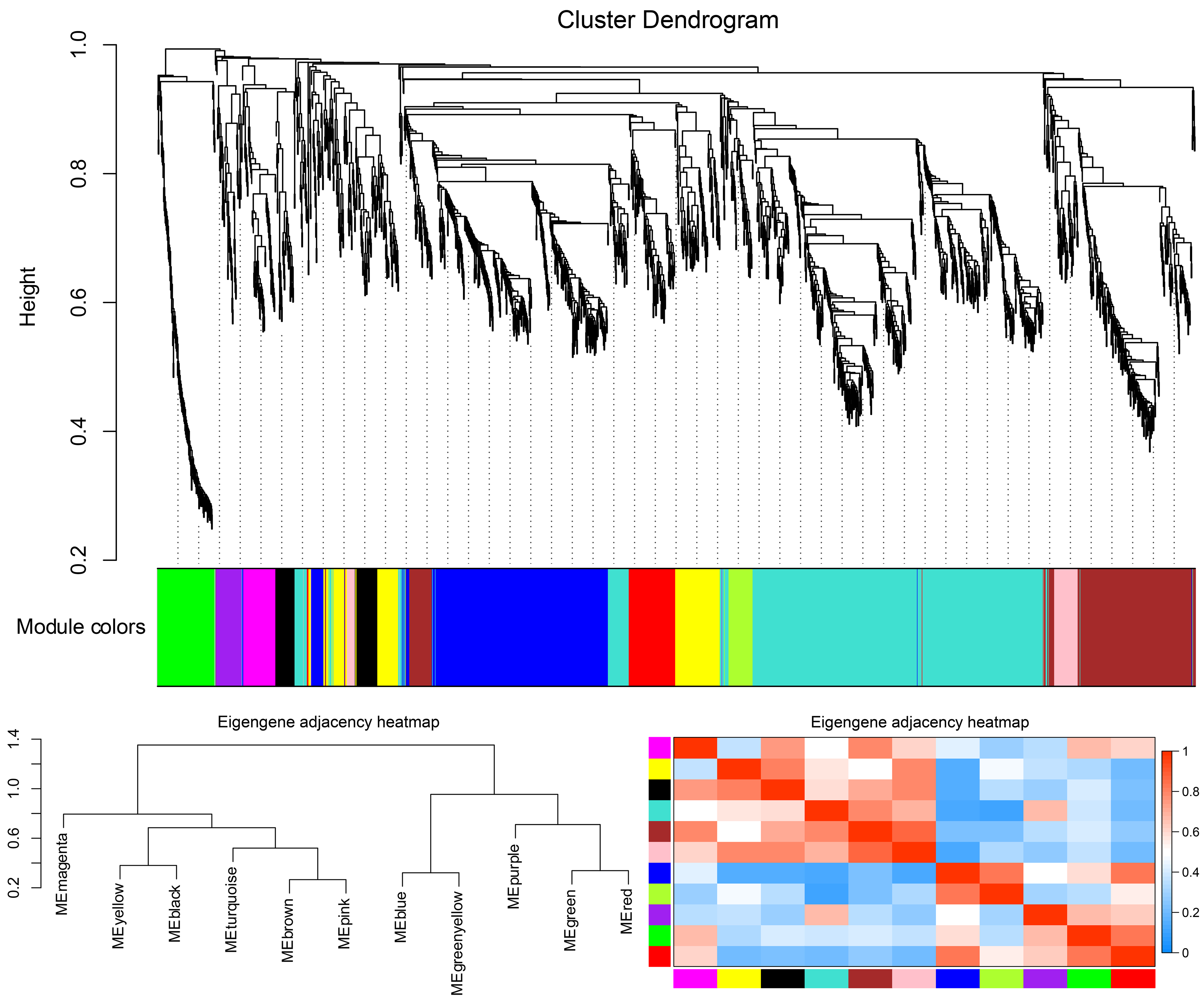


**Supplementary Figure 4** Hierarchical clustering dendrogram of module and module eigengenes and heatmap plot of the adjacencies in the eigengene network.


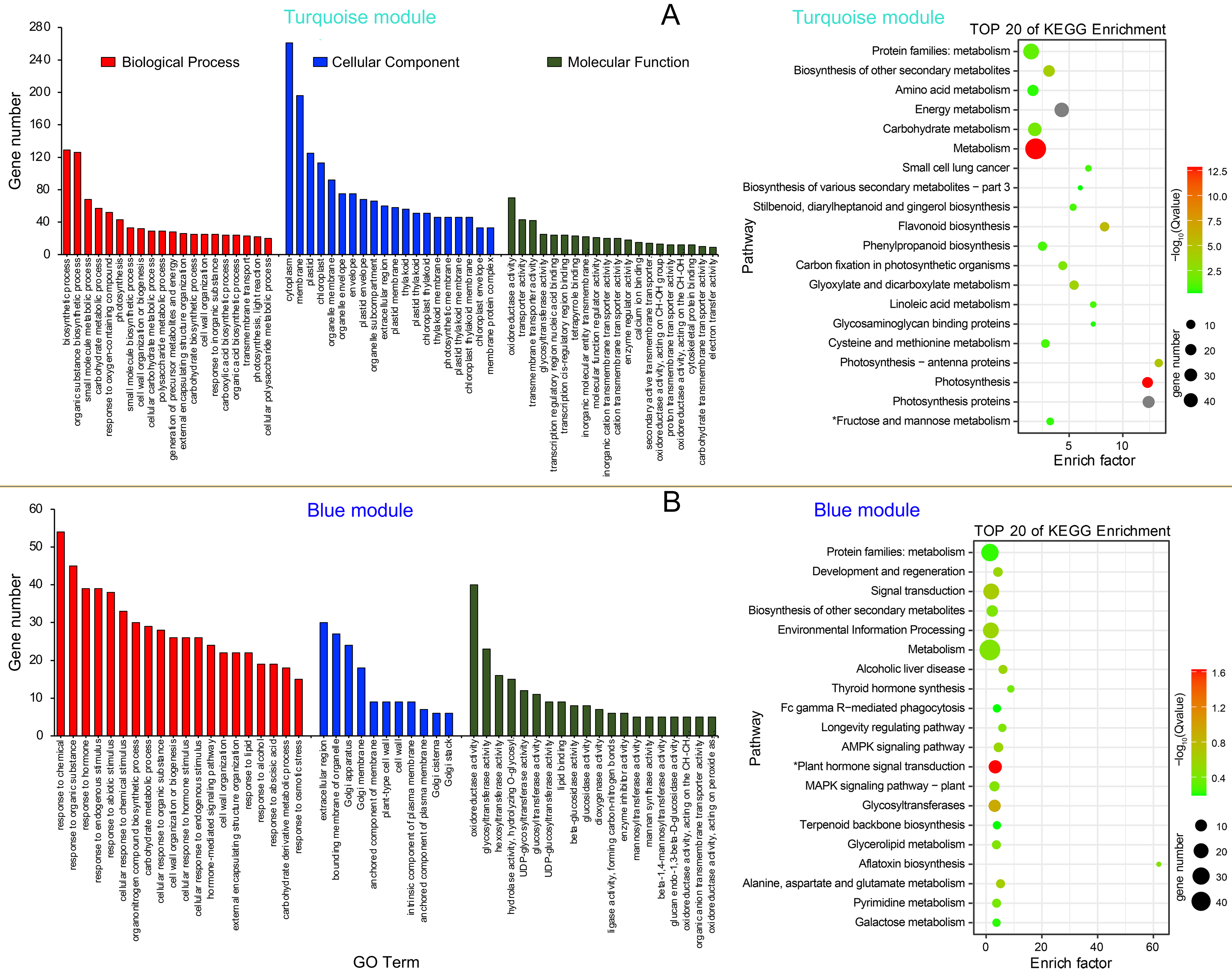


**Supplementary Figure 5** GO and KEGG enrichment analysis of the genes in module Turquoise (**A**) and Blue (**B**).


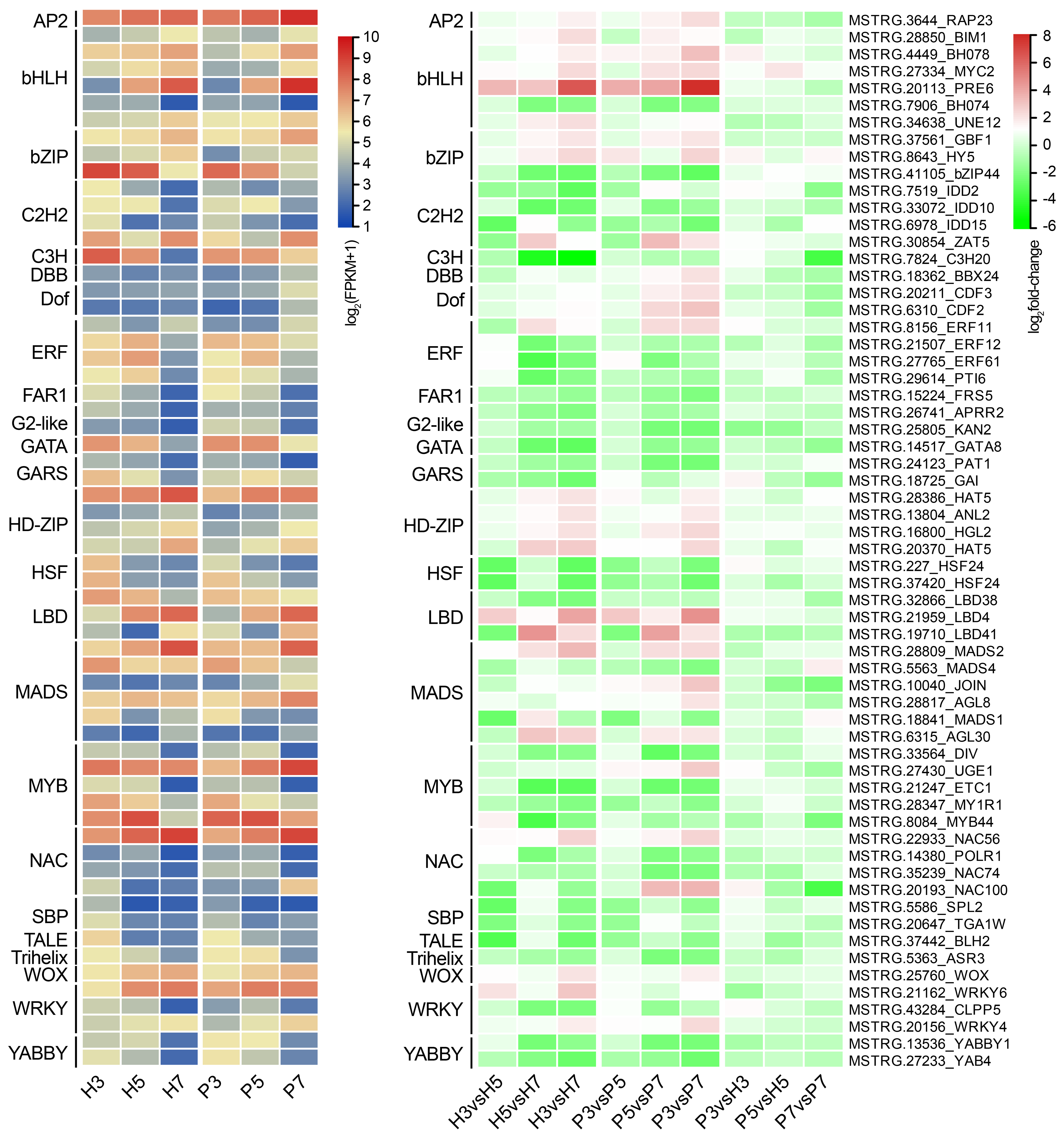


**Supplementary Figure 6** Differential expression of co-expressed transcription factors in Chinese cherry. The color scales indicate expression levels as log_2_(FPKM + 1) and log_2_fold-change values, respectively.
